# GlioSurvQNet: A DuelContextAttn DQN Framework for Brain Tumor Prognosis with Metaheuristic Optimization

**DOI:** 10.3390/diagnostics15182304

**Published:** 2025-09-11

**Authors:** M. Renugadevi, Venkateswarlu Gonuguntla, Ihssan S. Masad, G. Venkat Babu, K. Narasimhan

**Affiliations:** 1 School of Electrical and Electronics Engineering, SASTRA Deemed University, Thanjavur 613401, India; renugadevi@ece.sastra.ac.in (M.R.); venkatbabu@ece.sastra.edu (G.V.B.); 2 Symbiosis Centre for Medical Image Analysis, Symbiosis International (Deemed University), Pune 412115, India; 3 Department of Electrical and Computer Engineering, Gulf University for Science and Technology (GUST), Hawally 32093, Kuwait; masad.i@gust.edu.kw; 4 Department of Biomedical Systems and Informatics Engineering, Hijjawi Faculty for Engineering Technology, Yarmouk University, Irbid 21163, Jordan; 5 GUST Engineering & Applied Innovation Research Center (GEAR), Gulf University for Science and Technology (GUST), Hawally 32093, Kuwait

**Keywords:** brain tumor classification, Dueling DQN, metaheuristic optimization, radiomics, reinforcement learning, survival prediction

## Abstract

**Background/Objectives:** Accurate classification of brain tumors and reliable prediction of patient survival are essential in neuro-oncology, guiding clinical decisions and enabling precision treatment planning. However, conventional machine learning and deep learning methods often struggle with challenges such as data scarcity, class imbalance, limited model interpretability, and poor generalization across diverse clinical settings. This study presents GlioSurvQNet, a novel reinforcement learning-based framework designed to address these limitations for both glioma grading and survival prediction. **Methods:** GlioSurvQNet is built upon a DuelContextAttn Deep Q-Network (DQN) architecture, tailored for binary classification of low-grade vs. high-grade gliomas and multi-class survival prediction (short-, medium-, and long-term categories). Radiomics features were extracted from multimodal MRI scans, including FLAIR, T1CE, and T2 sequences. Feature optimization was performed using a hybrid ensemble of metaheuristic algorithms, including Harris Hawks Optimization (HHO), Modified Gorilla Troops Optimization (mGTO), and Zebra Optimization Algorithm (ZOA). Subsequently, SHAP-based feature selection was applied to enhance model interpretability and robustness. **Results:** The classification module achieved the highest accuracy of 99.27% using the FLAIR + T1CE modality pair, while the survival prediction model attained an accuracy of 93.82% with the FLAIR + T2 + T1CE fusion. Comparative evaluations against established machine learning and deep learning models demonstrated that GlioSurvQNet consistently outperformed existing approaches in both tasks. **Conclusions:** GlioSurvQNet offers a powerful and interpretable AI-driven solution for brain tumor analysis. Its high accuracy and robustness make it a promising tool for clinical decision support in glioma diagnosis and prognosis.

## 1. Introduction

Gliomas are one of the most common types of brain tumors [1]. Their behavior and patient outcomes can vary greatly depending on whether the tumor is low-grade (LGG) or high-grade (HGG). Low-grade gliomas usually grow slowly and offer better survival chances, while high-grade gliomas, such as glioblastomas, are much more aggressive and harder to treat. Because of this, identifying the tumor grade early and predicting how long a patient is likely to survive are important steps in planning effective treatment [2].

MRI scans are widely used to examine brain tumors, and radiomics allows us to extract useful information from these images by turning them into quantitative features. In the past, machine learning methods like support vector machines and random forests have been used to classify glioma types based on these features. While these approaches can work well, they often rely too much on carefully selected features and may not perform consistently when data are limited or unbalanced [3,4].

More recently, deep learning models such as convolutional neural networks (CNNs) and transformers have become popular because they can learn patterns directly from imaging data [5,6]. These models have shown good results in brain tumor detection, segmentation, and even survival prediction.

However, despite their effectiveness, machine learning and deep learning methodologies in medical image analysis still face critical challenges, including the requirement for large labeled datasets, the high dimensionality of radiomics features, limited generalization to unseen data, and insufficient interpretability, ultimately leading to suboptimal predictive performance [7]. Reinforcement learning (RL) offers a promising alternative by addressing several of these limitations. Unlike traditional supervised learning, which relies on static training examples, RL algorithms learn through trial-and-error interactions with an environment, receiving feedback in the form of rewards [8]. This reward-driven, sequential decision-making process enables RL models to dynamically adapt to changing conditions, integrate multi-step reasoning, and optimize long-term outcomes. Such capabilities make RL particularly well-suited for complex medical imaging tasks, including diagnosis and prognosis, where decisions often depend on cumulative evidence over multiple steps. While RL has shown potential in broader medical imaging applications [9,10,11], its application to brain tumor classification and survival prediction, especially in integrating imaging and clinical data, remains largely unexplored [12].

Another important step in building accurate models is selecting the right features. Radiomics often produces a large number of features, many of which may not be useful. Traditional selection methods like LASSO or recursive feature elimination work, but they can struggle when the data are high-dimensional or unbalanced [13]. Metaheuristic algorithms such as Harris Hawks Optimization (HHO), Modified Gorilla Troops Optimization (mGTO), and Zebra Optimization Algorithm (ZOA) offer more flexible ways to choose important features [14]. Still, few studies have combined these methods or applied them with a focus on medical relevance and interpretability.

This study introduces GlioSurvQNet, a reinforcement-learning-based framework for glioma grade classification and survival prediction. At the heart of this system is a custom deep RL model called DuelContextAttn DQN, which uses an attention mechanism to focus on the most informative features during learning. To improve the model’s reliability and reduce unnecessary complexity, we apply an ensemble of metaheuristic optimization techniques to select key radiomics features, followed by SHAP-based filtering to enhance interpretability.

The framework is evaluated on the BraTS2020 dataset, which includes multimodal MRI scans and clinical information. GlioSurvQNet is trained to perform two tasks: (1) classify tumors as LGG or HGG, and (2) categorize patients into short, medium, or long-term survival groups. Our results show that the model performs strongly in both tasks while offering better adaptability and explanation than traditional approaches.

- A reinforcement learning-based model that can handle both tumor grading and survival prediction tasks.
- A novel deep Q-network with attention (DuelContextAttn DQN) that improves decision-making using radiomics data.
- A robust feature selection pipeline combining multiple metaheuristic algorithms with SHAP for clinical interpretability.

This work shows the potential of combining reinforcement learning, optimized feature selection, and explainable AI to support accurate and more reliable brain tumor analysis. The paper is structured as follows: Section 2 elaborates on the proposed framework and its implementation. Section 3 discusses the experimental results and compares them with existing literature. Finally, Section 4 summarizes the key findings and outlines avenues for future research.

## 2. Materials and Methods

**Figure 1:**
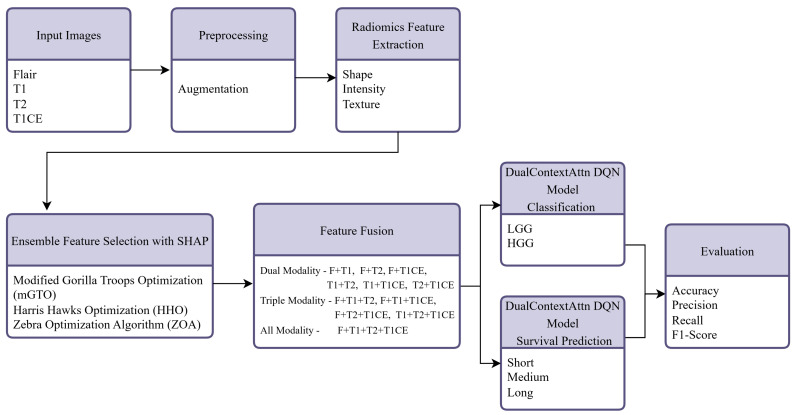
The overall process of the GlioSurvQNet for the classification and survival prediction using DuelContextAttn DQN with Metaheuristic Feature Selection.

This section details the development and evaluation of the proposed GlioSurvQNet framework. The model is designed to perform two tasks: (1) classify glioma tumors as LGG or HGG, and (2) predict overall survival duration in three categories. The overall workflow, including radiomics feature extraction from multimodal MRI scans, ensemble-based feature selection, and reinforcement-learning-driven classification and survival prediction, is illustrated in Figure 1, providing a comprehensive overview of the proposed GlioSurvQNet framework.

### 2.1. Dataset Description

This Multimodal Brain Tumor Segmentation dataset is essential for tasks such as tumor segmentation, classification, and survival analysis [15,16]. The dataset encompasses pre-operative MRI scans in four distinct modalities, including T1-weighted, T2-weighted, T1-weighted with contrast enhancement (T1-CE), and fluid-attenuated inversion recovery (FLAIR) pre-operative scans. The dataset consists of 369 patient cases, which include 76 cases of LGG and 293 cases of HGG, providing a comprehensive array of multimodal MRI scans that are crucial for precise classification. Along with the MRI scans, clinical data, including patient age, survival duration (in days), and resection status, are available for 235 cases to support overall survival prediction.

### 2.2. Feature Extraction and Selection

Radiomics feature extraction is a crucial step in quantitative medical imaging analysis, enabling the derivation of numerous informative features that support diagnosis, prognosis, and clinical research. To address the data imbalance between LGG and HGG cases, various augmentation strategies were employed exclusively for LGG cases. These included spatial alterations such as directional flipping, rotational transformations, and controlled noise perturbations, all designed to expand the dataset while retaining anatomical validity synthetically. As a result, the LGG class was increased to 304 samples, producing a balanced dataset comprising 595 images (304 LGG and 291 HGG) for downstream classification tasks. Importantly, the data split was performed with unique patient identifiers to ensure that no augmented versions of the same patient appeared in both the training and testing sets, thereby preventing any risk of data leakage.

Radiomics features were extracted using PyRadiomics (radiomics 3.1.0). Prior to feature extraction, MRI volumes were normalized per volume using z-score normalization within the brain mask. Volumes were resampled to isotropic voxels of 1×1×1 mm using a B-spline interpolator. Texture features were computed after fixed-width discretization with a bin width of 25. Comprehensive extraction details are fully documented, and the IBSI-compliant YAML configuration file is provided in the Supplementary Materials. Segmentation masks were directly obtained from the BraTS ground-truth annotations, and radiomics features were extracted in 3D from the full volumetric ROIs, covering the whole tumor (WT), tumor core (TC), and enhancing tumor (ET). Radiomics features capture geometric (e.g., volume, sphericity), statistical (e.g., mean, skewness), and spatial (e.g., GLCM, GLRLM) characteristics of the tumor. A total of 105 features were extracted, as summarized in Table 1. Given the high dimensionality and potential redundancy in radiomics data, a two-stage feature selection process was employed. First, three metaheuristic algorithms, namely HHO, mGTO, and ZOA, were used to select informative features. Each algorithm explores different regions of the feature space and selects feature subsets that yield better classification or survival prediction results. Second, SHAP (SHapley Additive exPlanations) filtering was applied to enhance interpretability by eliminating features with consistently low importance, ensuring that only those contributing meaningfully to model predictions were retained.

#### 2.2.1. Harris Hawks Optimization Algorithm

HHO is a nature-inspired optimization algorithm that models the hunting strategies of Harris hawks [17]. In feature selection, each hawk represents a subset of features, and the algorithm adaptively balances exploration and exploitation to identify the most relevant features. By simulating different attack strategies, HHO efficiently reduces feature dimensionality while maintaining or improving model performance.

#### 2.2.2. Modified Gorilla Troops Optimization Algorithm

mGTO is an enhanced variant of the Gorilla Troops Optimization (GTO) algorithm, which draws inspiration from the social structure and movement strategies of gorilla troops [18]. It models the leadership behavior of dominant silverbacks to guide the search process. The modified version improves the balance between exploration and exploitation, making it more effective for solving complex optimization tasks such as feature selection.

#### 2.2.3. Zebra Optimization Algorithm

ZOA is a bio-inspired metaheuristic based on the social behavior and movement patterns of zebras in the wild [19]. It simulates how zebras use group dynamics, vigilance, and coordinated movement to explore their environment efficiently. In feature selection, ZOA searches for optimal subsets by balancing diversification and intensification, effectively reducing redundancy while retaining informative features.

#### Feature Selection

The hyperparameters for the metaheuristic feature selection algorithms (HHO, mGTO, ZOA) are summarized in Table 2. Each algorithm was configured with a population size of 20 and executed for 50 iterations. The search space was defined as a binary vector across 105 features, where each element corresponds to a feature’s selection status (1 for selected, 0 for not selected). The optimization was framed as a minimization problem, with the objective function evaluating classification accuracy using a linear SVM. This classifier was trained exclusively on the training folds of a stratified k-fold cross-validation scheme to prevent any data leakage. Each algorithm was executed 10 times with different random seeds, and the average accuracy was reported. Following the execution of individual optimization algorithms, each method generated a binary mask identifying informative features. To enhance robustness and reduce individual algorithm bias, a majority voting scheme was applied across HHO, mGTO, and ZOA, as shown in Algorithm 1. Features selected by at least two algorithms were retained in the ensemble mask. A linear SVM was employed in the fitness function due to its suitability for high-dimensional, small-sample data. Both the majority voting and subsequent feature filtering steps were performed separately within each training fold to maintain strict independence from the test data.

**Figure 2:**
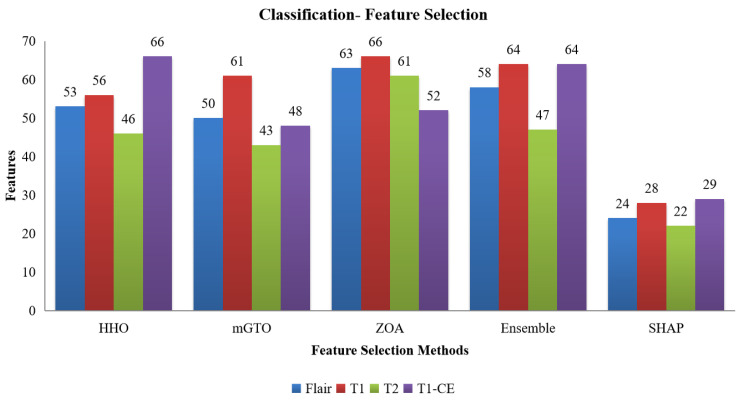
Number of features selected from the HHO, mGTO, ZOA, Ensemble Metaheuristic Optimization Algorithm, and SHAP for each modality used in Classification.

**Figure 3:**
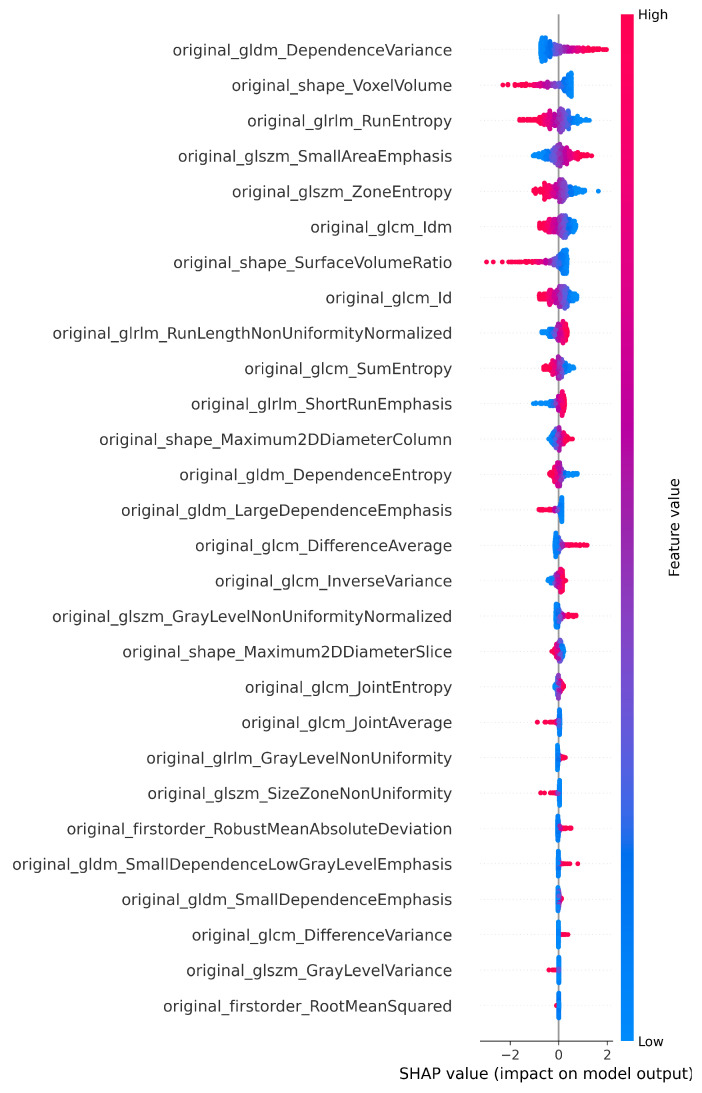
SHAP plot showing the selected features of T1 for the classification.

**Figure 4:**
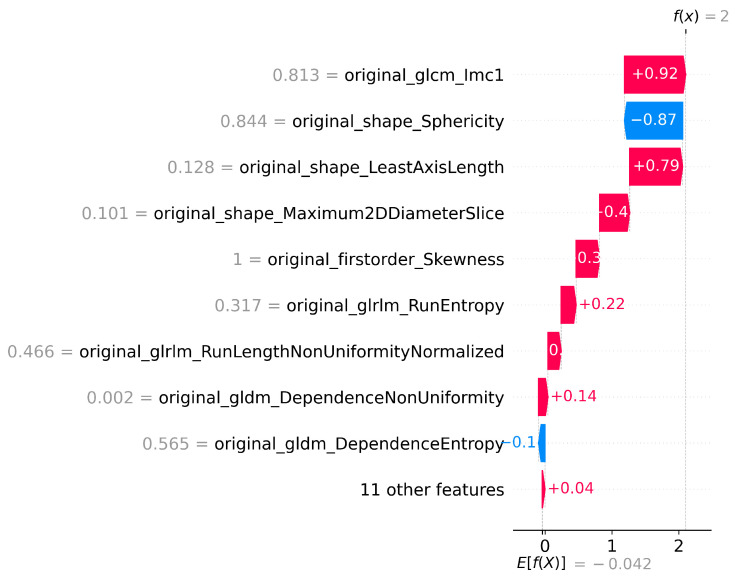
SHAP waterfall plot for T1 Features for LGG vs HGG classification.

The number of features selected by each method, including the ensemble and SHAP refinement, is summarized in Figure 2. Specifically, the ensemble method selected 58 features from Flair, 64 from T1, 47 from T2, and 64 from T1-CE. To ensure interpretability, SHAP was used to rank features, retaining only those contributing to 90% of the cumulative SHAP value [20]. This ensemble-SHAP strategy produced stable, discriminative, and explainable feature subsets across all MRI modalities, selecting 24, 28, 22, and 29 features from Flair, T1, T2, and T1-CE, respectively. The SHAP-selected features of T1 are visualized in Figure 3, illustrating the relative importance of each feature in differentiating between LGG (class 0) and HGG (class 1). The waterfall plot shown in Figure 4 illustrates how individual features push predictions toward either LGG or HGG, offering clinically interpretable insights into model decision-making. The identified T1 features correlate with known pathophysiological differences between LGG and HGG tumors, including heterogeneity, boundary sharpness, and intensity variations. This suggests that the model is capturing clinically relevant tumor characteristics rather than relying on spurious correlations.
**Algorithm 1** Ensemble Feature Selection with SHAP Refinement**Input:** Dataset *D* with features *X*, labels *y*, threshold θNormalize *X* and split into train/test sets**function** Fitness(solution)      Convert solution to binary mask      **if** no feature selected **then**            **return** 1.0      **end if**      Train SVM on selected features      **return** 1− accuracy**end function**Define a binary optimization problem with Fitness as objectiveRun HHO, mGTO, and ZOA to generate feature masksApply majority voting to obtain ensemble maskTrain SVM using features selected by ensemble maskCompute SHAP values; rank features by mean absolute valueSelect top features achieving θ cumulative SHAP contribution**Output:** Optimized feature subset $X_{\mathrm{SHAP}}$

#### Feature Fusion

After identifying modality-specific features using the ensemble-SHAP approach, feature fusion was performed to explore multi-modal integration strategies. This fusion was performed at three levels: dual, triple, and four-modality combinations. In the dual-modality setup, six combinations were evaluated: Flair + T1, Flair + T2, Flair + T1CE, T1 + T2, T1 + T1CE, and T2 + T1CE. These pairs allowed the model to benefit from complementary structural and contrast-enhanced information. Building upon this, triple-modality fusion was explored through four key combinations: Flair + T1 + T2, Flair + T1 + T1CE, Flair + T2 + T1CE, and T1 + T2 + T1CE. These combinations integrated a broader context of anatomical and pathological features, enriching the model’s understanding of tumor heterogeneity. Finally, a four-modality fusion incorporating all sequences, Flair, T1, T2, and T1CE, was performed to capture the full range of tumor characteristics. This step aimed to leverage complementary information across MRI modalities for improved glioma classification performance.

### 2.3. DuelContextAttn DQN RL Framework

The reinforcement learning environment was implemented using the gym.Env framework and customized to simulate interactions between the agent and the dataset for brain tumor classification and survival prediction. The environment includes the following key components:

State: Each state represents a set of extracted features corresponding to a patient’s medical data, forming a unique feature vector for an individual sample.

Action Space:

- LGG/HGG Classification: The action space is discrete with two possible actions: action 0 and action 1. Action 0 corresponds to predicting an LGG, while action 1 represents a prediction of HGG.
- Survival Classification: The action space consists of three discrete actions: 0, 1, and 2, corresponding to predictions of short-term, medium-term, and long-term survival, respectively.

Reward Function: The environment provides feedback in the form of rewards after each action. A reward of +1 is granted if the agent’s prediction matches the ground truth label, while a penalty of −1 is given for incorrect predictions. This reward mechanism encourages the agent to improve its policy over time for better predictive accuracy.

Agent: The agent is based on a Dueling Deep Q-Network architecture, specifically a DuelContextAttn DQN variant. It employs an ϵ-greedy policy to balance exploration and exploitation, maintains a replay buffer to store past experiences, and updates its parameters by minimizing the mean squared error between predicted and target Q-values.

Model: The DuelContextAttn DQN architecture includes shared hidden layers followed by two separate output streams: one for estimating the state-value function and another for computing action advantages. A contextual attention mechanism dynamically assigns learnable weights to modulate the fusion of these two streams, enabling more accurate Q-value estimation.

#### 2.3.1. DuelContextAttn DQN

DuelContextAttn DQN is an enhanced version of the traditional DQN algorithm used in the realm of reinforcement learning [21]. The DQN is an advanced reinforcement learning algorithm that merges deep learning techniques with Q-learning to effectively manage high-dimensional state spaces [22]. In conventional Q-learning, state-action value pairs are organized in a Q-table, which works well for smaller state spaces. However, as the complexity of problems increases and the number of states grows, maintaining a comprehensive table becomes unfeasible. DQN addresses this challenge by employing a deep neural network to approximate Q-values, making it adept at navigating high-dimensional environments. The states are given as input to the network, which outputs the corresponding Q-values. For a given state *s* and action *a*, the network approximates the Q-function, denoted as Q(s,a), by providing Q(s,a;θ) as output, where θ represents the network parameters.

DQN employs two essential techniques to enhance training stability: experience replay and the use of two Q-networks, namely the main Q-network and the target network. Experience replay functions as a memory buffer, storing past experiences $\left(s,a,r,s^{\prime }\right)$ and randomly sampling them during training. This process helps to reduce correlations and enhance learning efficiency. The target network maintains a periodically updated copy of the main Q-network, which stabilizes learning by mitigating rapid fluctuations in Q-value estimates.

The target network’s parameters $\left(\theta^{-}\right)$ are updated at each time step using the following Equations (1) and (2):

(1) $$y_{t}^{DQN}=r_{t+1}+\gamma Q\left(s_{t+1},a^{\prime };\theta_{t}^{-}\right)$$

where γ is the discount factor and $\theta^{-}$ represents the target network parameters.

The loss function is:

(2) $$L_{i}\left(\theta_{i}\right)=E_{\left(s,a,r,s^{\prime }\right)}\left[{\left(y_{t}^{DQN}-Q\left(s,a;\theta_{i}\right)\right)}^{2}\right]$$

Additionally, DQN implements an ϵ-greedy policy, where the agent begins by exploring the environment with random actions and gradually shifts towards exploiting the action with the highest predicted Q-value. By integrating deep learning for function approximation and reinforcement learning for decision-making, DQN allows agents to learn optimal policies from raw state inputs, positioning it as a robust solution for complex control tasks.

Dueling DQN is an improvement over DQN to tackle the challenge of overestimation bias, which occurs when the Q-network overestimates action values, leading to suboptimal policies. In addition to the standard DQN approach, Dueling DQN introduces an enhanced architecture that consists of two essential components: the Value function and the Advantage function. This dual approach allows for a more effective computation of Q-values, leading to enhanced stability and efficiency in the learning process.

#### 2.3.2. Architecture Overview

**Figure 5:**
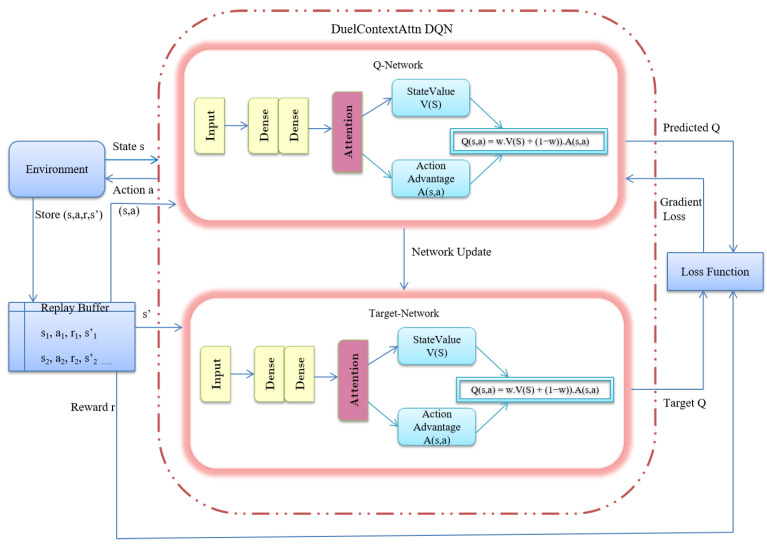
Architecture of the DuelContextAttn DQN model for Classification and Survival Prediction.

The DuelContextAttn DQN builds upon the foundation of Dueling DQN by introducing a context-aware attention mechanism that dynamically adjusts the relative importance of the value and advantage streams. The architecture of the DuelContextAttn DQN model for Classification and Survival Prediction is shown in Figure 5. In the traditional Dueling DQN framework, the Q-value is computed as the sum of a value function V(s) and an advantage function A(s,a):

(3) $$Q\left(s,a\right)=V\left(s\right)+\left(A\left(s,a\right)-\frac{1}{\left|A\right|}\sum_{a^{\prime }}A\left(s,a^{\prime }\right)\right)$$

This formulation implicitly assumes a fixed weighting between the value and advantage components across all states and actions. However, such an assumption may not be optimal, especially in high-dimensional domains like medical imaging, where the relative importance of features can vary significantly depending on the context.

To address this limitation, the DuelContextAttn DQN introduces a learnable attention-based fusion weight w(s) that dynamically modulates the contribution of the value and advantage streams based on the input state. This weight is derived from high-level features extracted from the input, enabling the model to better capture the context of each state-action pair. The key components of the architecture are

Value Stream (V(s)): Represents the overall value of a given state *s*, derived from attention-refined features. It reflects the expected long-term reward obtainable from that state, irrespective of the action taken.

Advantage Stream (A(s,a)): Captures the relative importance of selecting action *a* in state *s* compared to other possible actions. The attention mechanism enhances this stream by focusing on features most relevant to distinguishing among actions.

Attention Fusion Weight (w(s)): A learnable scalar function that assigns a dynamic weight to the value and advantage components. This weight is computed from the contextual features of the state, allowing flexible Q-value composition tailored to the specific input.

The final Q-value is computed as a convex combination of the value and normalized advantage streams:

(4) $$\begin{array}{cc} Q(s,a;\theta )= & \;w(s)\cdot V(s)\\ \; & +\left(1-w(s)\right)\cdot \left(A\left(s,a\right)-\frac{1}{\left|A\right|}\sum_{a^{\prime }}A\left(s,a^{\prime }\right)\right) \end{array}$$

This adaptive fusion mechanism enables the network to prioritize different aspects of the decision process depending on the contextual information present in each state, which is especially beneficial in medical imaging applications where subtle differences can be critical. The overall functioning of DuelContextAttn DQN is illustrated in Algorithm 2.

Each episode begins with a reset of the environment, enabling the agent to interact until it has completed a full traversal of the dataset. For every time step, the agent selects an action according to the current state using the epsilon-greedy policy. The environment then provides the subsequent state, the reward received, and an indication of whether the episode has ended. This interaction is stored in the agent’s memory, which is later used for replay to refine the Q-values. The agent’s performance is evaluated at the end of each episode in terms of accuracy and reward. The training process spans 50 episodes representing complete passes through the dataset, enabling iterative policy refinement for classification using radiomics features. The agent utilizes the Adam optimizer with a learning rate of 0.001 to ensure efficient gradient updates and stable learning. The mini-batches of size 16 are drawn from the replay buffer for batch learning. The Mean Squared Error (MSE) loss function is applied to measure the discrepancy between predicted and target Q-values. The training parameters used to train the DuelContextAttn DQN model are listed in Table 3.
**Algorithm 2** DualContextAtt DQN Algorithm**Step 1: Initialize**Initialize Q-network and target Q-network with dueling architecture and context-aware self-attention.Initialize replay buffer *D*.**for** each episode **do**      Reset environment and observe initial state *s*.      **for** each time step **do**            Select action $a_{t}$ using ϵ-greedy:$$a_{t}=\arg\max_{a}Q\left(s,a;\theta \right)$$            Execute $a_{t}$, observe reward $r_{t}$ and next state $s_{t+1}$.            Store $\left(s_{t},a_{t},r_{t},s_{t+1}\right)$ in buffer *D*.            Sample mini-batch of transitions $\left(s,a,r,s^{\prime }\right)$ from *D*.            **Step 2: Compute Target Q-values**$$y_{i}=\left\{\begin{array}{cc} r_{i}+\gamma \max_{a^{\prime }}Q^{\prime }\left(s^{\prime },a^{\prime };\theta^{-}\right), & \mathrm{if}\;\mathrm{not}\;\mathrm{terminal}\\ r_{i}, & \mathrm{if}\;\mathrm{terminal} \end{array}\right.$$            **Step 3: Compute Predicted Q-values with Attention Fusion**            Compute value stream V(s;θ)            Compute advantage stream A(s,a;θ)            Compute attention fusion weight w(s;θ)            Fuse:$$Q\left(s,a;\theta \right)=w\cdot V\left(s\right)+\left(1-w\right)\cdot \left(A\left(s,a\right)-\frac{1}{\left|A\right|}\sum_{a^{\prime }}A\left(s,a^{\prime }\right)\right)$$            **Step 4: Compute Loss (MSE)**$$L\left(\theta \right)=\frac{1}{N}\sum_{i\in N}{\left(Q_{\theta }\left(s_{i},a_{i}\right)-Q_{\theta }^{\prime }\left(s_{i},a_{i}\right)\right)}^{2}$$
where$$Q_{\theta }^{\prime }=R\left(s_{t},a_{t}\right)+\gamma \max_{a_{i}^{\prime }}Q_{\theta }\left(s_{i}^{\prime },a_{i}^{\prime }\right)$$            **Step 5: Update Parameters**            Perform gradient descent on L(θ)            Update target network parameters: $\theta^{-}\leftarrow \theta$ periodically      **end for****end for****Step 6: Repeat Until Convergence**Repeat steps until convergence or maximum episodes.

### 2.4. Survival Prediction

**Figure 6:**
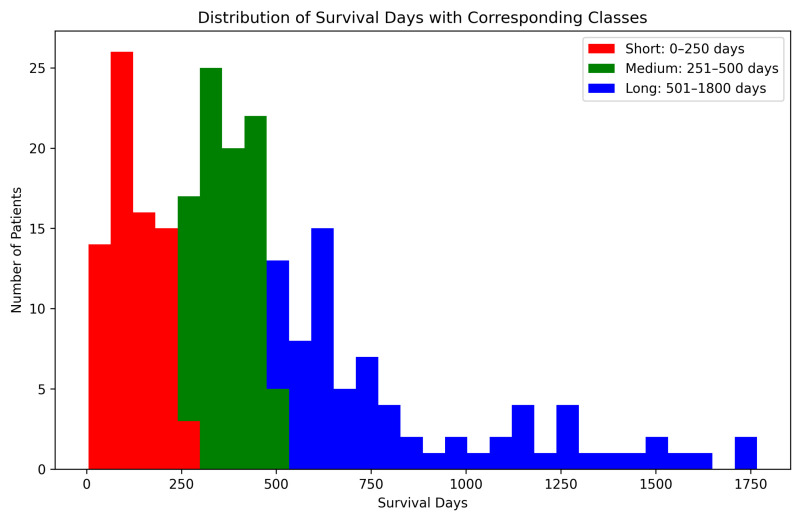
Histogram view for the survival class category short (0–250 days), medium (251–500 days), and long (501–1800 days).

The objective of survival prediction is to estimate the overall survival (OS) duration of patients using pre-treatment multimodal MRI scans along with relevant clinical variables. A total of 235 cases were analyzed and categorized into three survival classes: short (0–250 days), medium (251–500 days), and long (501–1800 days), consisting of 75, 86, and 74 cases, respectively. The distribution of these classes is illustrated in Figure 6. To address class imbalance and enrich the training dataset, data augmentation techniques were applied. Each 3D MRI volume was augmented using a combination of spatial transformations, including random horizontal and vertical flips, 90-degree rotations, and Gaussian noise injection. These augmentations preserved the anatomical structure while increasing variability and improving model generalization. Following augmentation, the dataset expanded to 705 samples, with Class 0 comprising 225 samples, Class 1 with 258 samples, and Class 2 with 222 samples. Subsequently, 105 radiomic features were extracted from each MRI modality. These features were combined with clinical data, including patient age and survival duration, to enhance the model’s predictive capability.

**Figure 7:**
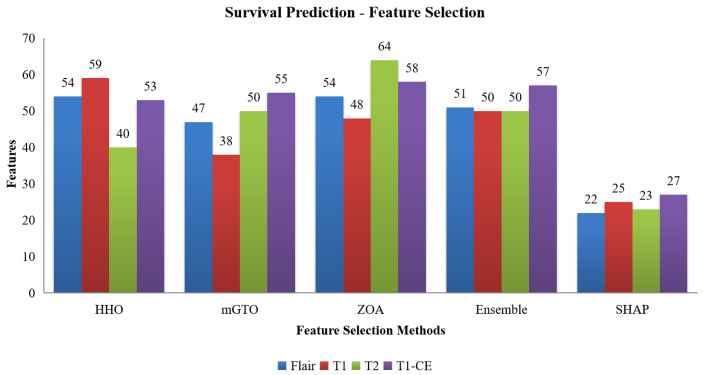
Number of features selected by HHO, mGTO, ZOA, Ensemble Metaheuristic Optimization Algorithm and SHAP for each modality used in survival prediction.

**Figure 8:**
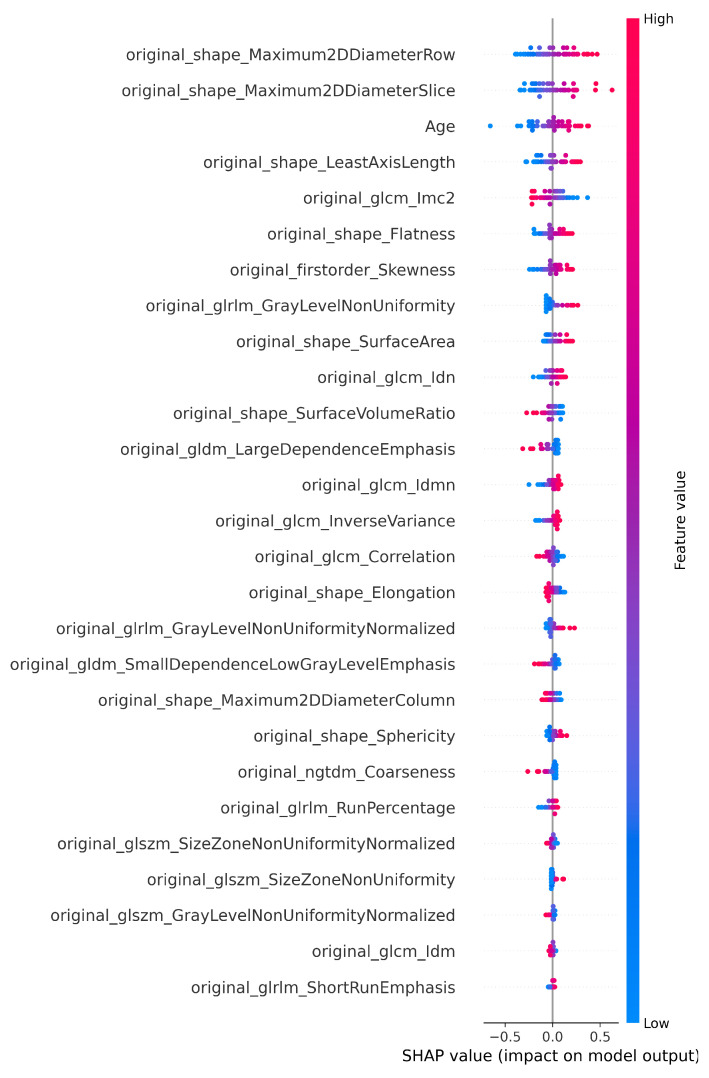
SHAP plot highlighting the contribution of T1-CE selected features to survival prediction.

**Figure 9:**
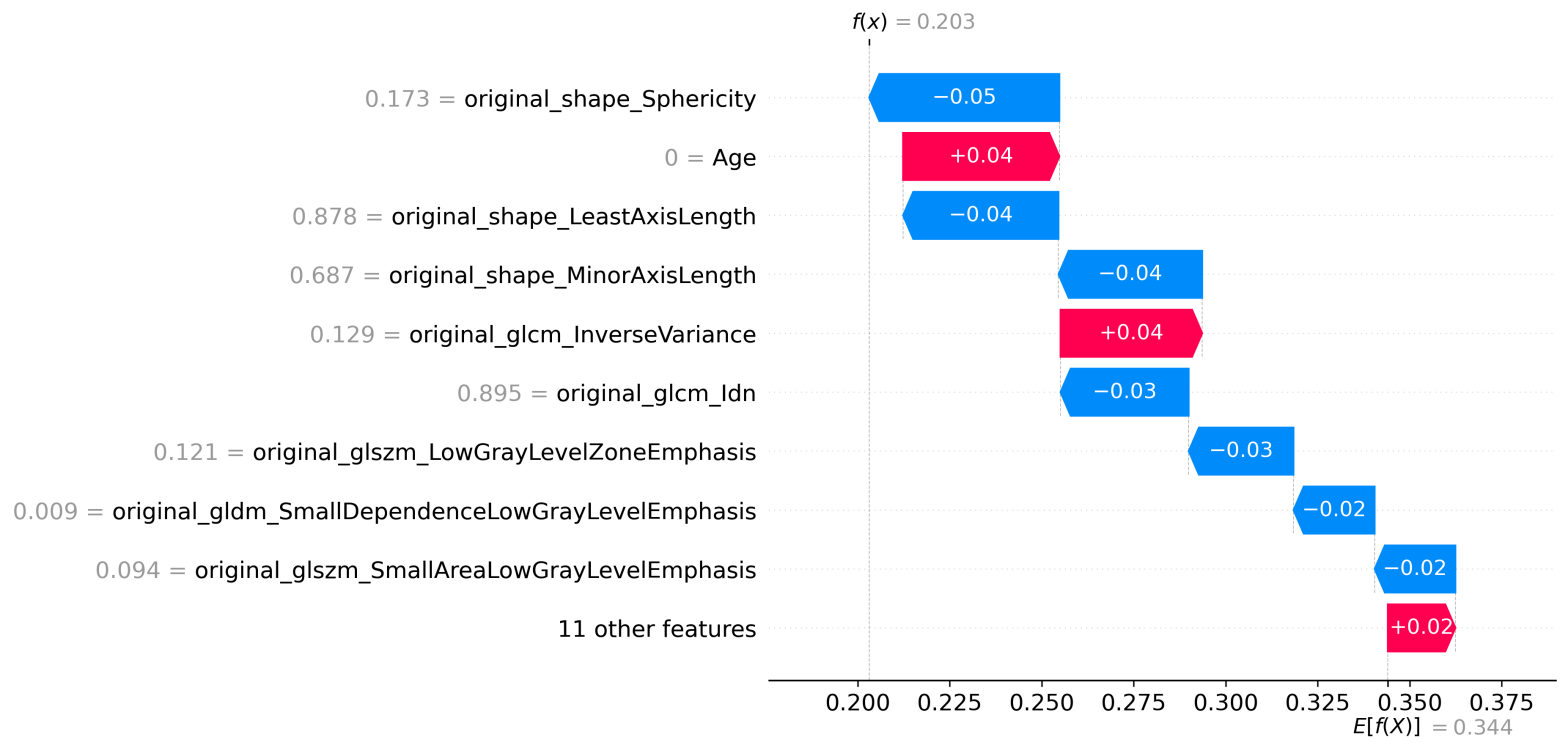
SHAP waterfall plot for T1CE (Survival prediction).

An ensemble of optimization algorithms, integrated with SHAP analysis, was applied for feature selection, enabling the identification of the most relevant features. As shown in Figure 7, 22 FLAIR features, 25 from T1, 23 from T2, and 27 from T1-CE were selected by the optimization algorithms, highlighting the variation in the relevance of features between imaging modalities. The summary plot shown in Figure 8 ranks the top predictors differentiating low, medium, and high survival groups. The waterfall plot shown in Figure 9 illustrates how these top-ranked features interact to predict a specific survival category. Collectively, the SHAP results provide transparent explanations for survival stratification, aligning with clinical understanding that contrast enhancement patterns are surrogates of tumor vascularity and progression.

## 3. Results and Discussion

### 3.1. Classification Performance and Comparative Study

The DuelContextAttn DQN model was trained using selected radiomics features for each MRI modality to classify LGG and HGG. All experiments were conducted on a workstation equipped with an Intel Core i7 CPU and an NVIDIA T1000 GPU with 4 GB memory, running a 64-bit Windows operating system. Each training run per modality consisted of 50 episodes, with an average episode time of approximately 610 s, resulting in a total runtime of ∼8.5 h per modality. A total of 595 patient cases were included for glioma grading, and the dataset was split into 80% for training and 20% for testing. This resulted in 476 samples for training and 119 for testing, ensuring a balanced evaluation of model performance across both phases. For robust performance estimation, 5-fold cross-validation was applied on the training set, and mean ± standard deviation across folds is reported. Hyperparameter tuning was conducted to optimize model performance, focusing on γ (Gamma), ϵ (Epsilon), $\epsilon_{\min}$ (Epsilon_min), and $\epsilon_{\mathrm{decay}}$ (Epsilon_decay). An ablation study was executed, exploring different combinations of these parameters to improve accuracy and minimize episode duration. The optimal performance was recorded with γ=0.99, ϵ=0.5, $\epsilon_{\min}=0.01$, and $\epsilon_{\mathrm{decay}}=0.995$, yielding an accuracy of 99% and an average episode time of 617.26 s, as detailed in Table 4.

Table 5 summarizes the 5-fold cross-validated classification performance of the model using individual and fused MRI modalities, reporting mean ± standard deviation with 95% CI (in parentheses) for precision, recall, F1-score, and accuracy [23]. Among the single modalities, T1 achieved the highest accuracy (99.02 ± 0.08%), demonstrating its superior ability to capture tumor-specific structural details, followed closely by T2 and Flair, both contributing substantially to classification outcomes. Fusion of modalities consistently enhanced performance, indicating that integrating complementary information strengthens discriminative power. Among the dual-modality combinations, F + T1CE and F + T2 delivered the best results, with accuracies of 99.27 ± 0.05% and 99.12 ± 0.08%, respectively, likely benefiting from the contrast-enhancing properties of T1CE and the edema sensitivity of Flair and T2.

**Figure 10:**
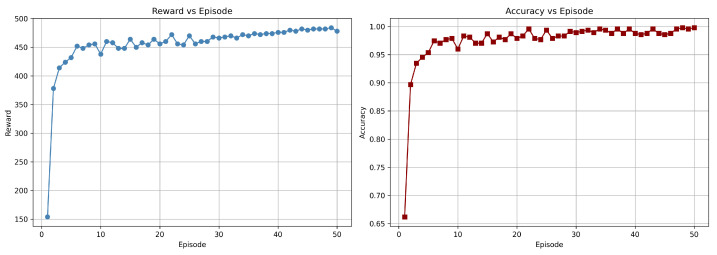
Training graph showing the progress of rewards and accuracy per episode for the DuelContextAttn DQN model in LGG/HGG classification using Flair + T1CE modality.

In triple-modality combinations, F + T1 + T1CE and F + T2 + T1CE achieved high accuracies of 98.95 ± 0.03% and 98.74 ± 0.05%, respectively, highlighting the contribution of T1CE in enhancing tumor characterization when fused with other structural sequences. The four-modality fusion (F + T1 + T2 + T1CE) reached 98.32 ± 0.05%, slightly lower than the best dual- and triple-modality combinations. Statistical comparison between the dual-modality combination (Flair + T1CE) and the 4-modality fusion showed no significant improvement: McNemar’s test (stat = 3.0, *p* = 0.508) and DeLong’s test (AUC dual: 0.984, all: 0.979) confirmed similar misclassification patterns and comparable discriminative ability. Feature correlation analysis revealed that many dual-modality features were highly correlated with those in the 4-modality set, indicating redundancy. These results suggest that while dual-modality combinations capture complementary information effectively, adding more modalities can introduce redundant features, higher dimensionality, and noise, slightly reducing performance. Figure 10 shows the training curves for the DuelContextAttn DQN model, illustrating the progression of reward and accuracy across episodes for the Flair + T1CE modality. These plots highlight the agent’s learning behavior, showing consistent improvement in both cumulative rewards and classification accuracy.

**Figure 11:**
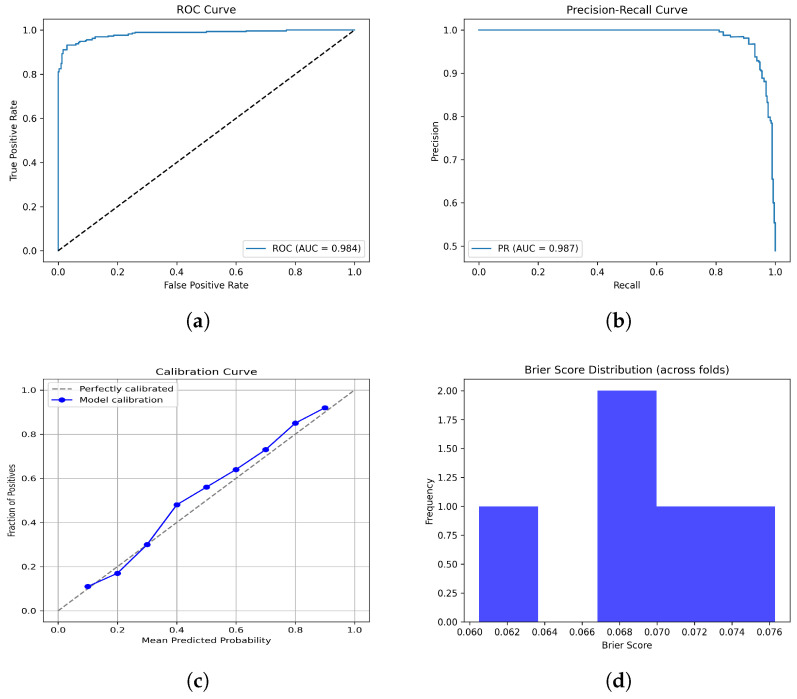
Performance since they have already been explained in the caption. Please confirm this revision. evaluation plots of the F + T1CE fusion model: (**a**) ROC curve, (**b**) PR curve, (**c**) Calibration curve, and (**d**) Brier scores.

Additionally, the ROC curve (Figure 11a, AUC = 0.984) and PR curve (Figure 11b, AUC = 0.987) demonstrate excellent discriminative ability with near-perfect precision across a wide recall range. The calibration curve (Figure 11c) shows good alignment between predicted probabilities and observed outcomes, indicating reliable probability estimates. Furthermore, the low Brier scores (Figure 11d) across folds (mean ≈ 0.067) confirm strong overall calibration. Together, these results highlight that the F + T1CE fusion model not only provides highly accurate classification but also yields well-calibrated and clinically trustworthy predictions.

**Figure 12:**
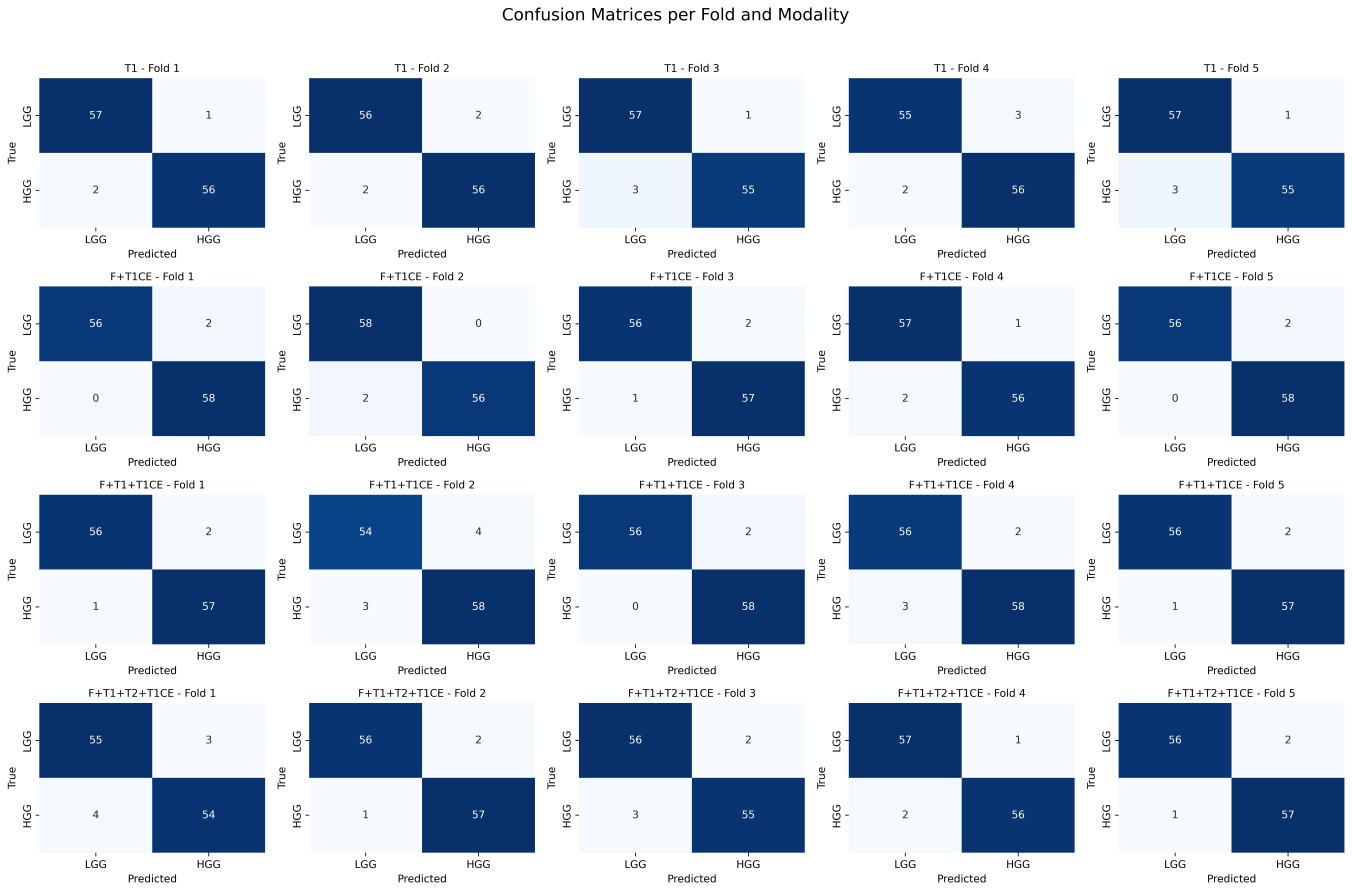
Confusion matrices per fold and modality for binary tumor grading classification. Each row corresponds to a modality combination (T1, F + T1CE, F + T1 + T1CE, F + T1 + T2 + T1CE), and each column corresponds to a cross-validation fold.

Figure 12 presents the confusion matrices obtained for the binary tumor grading task across five folds and multiple modality combinations. It is observed that all modalities achieve high true positive rates, with very few misclassifications, reflecting stable and robust performance across folds. Notably, the model demonstrates excellent classification performance for glioma classification using Flair + T1CE inputs, suggesting their effectiveness in discriminating between LGG and HGG.

To further strengthen our evaluation, the proposed method is compared with several strong conventional baselines to ensure fairness in implementation. All models were trained using the fused Flair + T1CE modality, which consistently yielded the highest accuracy in our experiments. The results, summarized in Table 6, clearly demonstrate that while conventional classifiers such as Logistic Regression, SVM, and ensemble tree-based methods (XGBoost, LightGBM, Random Forest, Gradient Boosting) achieve competitive performance, our proposed method significantly outperforms them across all metrics, particularly in terms of accuracy and F1-score.

Table 7 presents a comparative analysis of existing state-of-the-art models used for glioma classification on the BraTS dataset, showcasing their reported accuracy. Traditional machine learning methods such as Random Forest and SVM achieved high accuracies (up to 97.48%), while deep learning-based models like CNN, VGG, and hybrid approaches (e.g., TD-CNN-LSTM and DQL-TD) demonstrated further improvements, with the highest being 100% by Stember et al. using DQL-TD over 200 episodes.In contrast, the proposed DuelContextAttn DQN achieves a competitive accuracy of 99.27% in only 50 episodes, demonstrating both high predictive performance and faster convergence. This highlights the efficiency and robustness of the reinforcement learning strategy integrated with context-aware attention mechanisms.

### 3.2. Survival Prediction Performance and Comparative Study

The DuelContextAttn DQN model was developed to forecast the total number of survival days for patients, employing the same hyperparameters as those used in the classification task. The primary aim was to categorize tumor patients into three distinct survival groups: short, medium, and long. A total of 705 samples were used in this analysis, with an 80:20 split applied to create the training and testing datasets, respectively. This ensured that the model was trained on 564 cases and evaluated on 141, allowing for a robust assessment of its generalization capabilities. Table 8 summarizes the macro-precision, macro-recall, macro-F1-score, macro-AUC and accuracy for various combinations of MRI modalities used in survival prediction. Among the single modality models, T1CE achieved the highest accuracy (93.28%), emphasizing its strength in capturing contrast-enhanced tumor regions, which are often indicative of aggressive tumor behavior and therefore more predictive of survival outcomes.

In the dual modality group, the combination of FLAIR + T2 achieved the highest accuracy (93.71%), suggesting that these two structural modalities provide complementary information. FLAIR captures peritumoral edema, while T2 delineates both edema and tumor core, contributing to more reliable survival class predictions.For triple modality fusion, the best performance was observed with FLAIR + T2 + T1CE, reaching an accuracy of 93.82%. This shows that integrating contrast-enhanced imaging with structural and fluid-sensitive modalities significantly enhances the model’s ability to distinguish among short, medium, and long survival classes.The four modality fusion (FLAIR + T1 + T2 + T1CE) also performed well with an accuracy of 93.39%, though it did not exceed the best triple combination. This may be due to from feature redundancy, higher dimensionality, and an increased risk of overfitting. Moreover, the attention mechanism must distribute focus across multiple modalities, potentially diluting emphasis on the most informative features. Each modality also introduces its own noise and artifacts, so including too many can amplify unwanted signals rather than enhance the model’s discriminative power.

**Figure 13:**
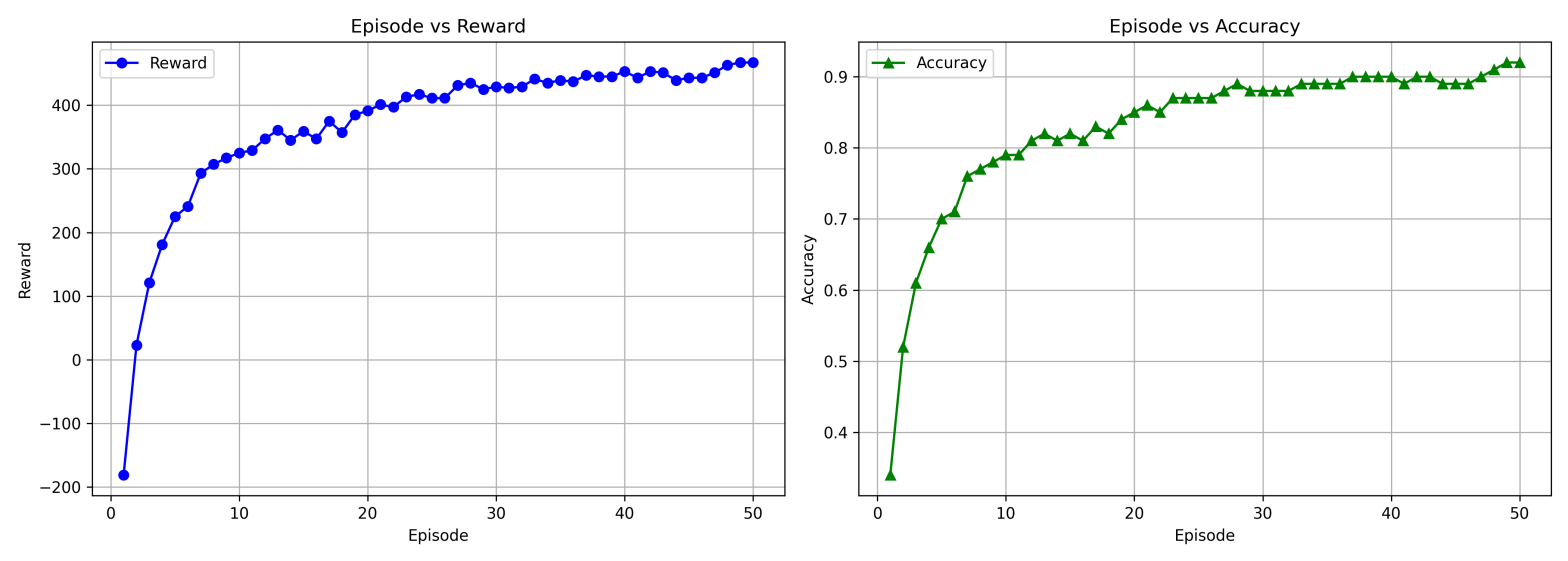
Training graph showing the progress of rewards and accuracy per episode for the DuelContextAttn DQN model in survival prediction using Flair + T1 + T1CE modality.

**Figure 14:**
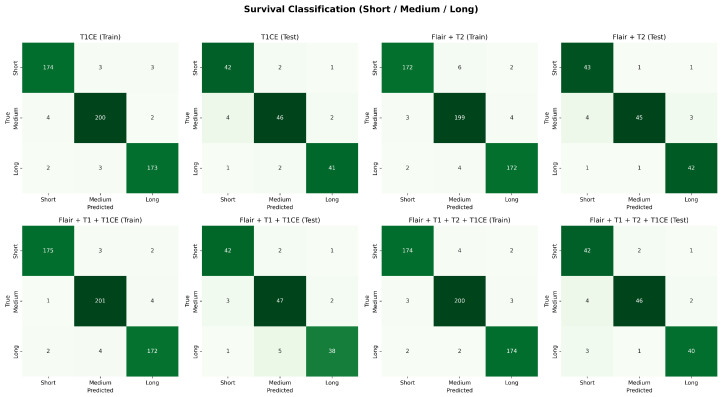
Confusion matrices for survival classification with best-performing modality combinations. The top row shows training results and testing results for T1CE, F + T2 and the bottom row shows for F + T1 + T1CE, and F + T1 + T2 + T1CE.

Figure 13 presents the learning curves for survival prediction using the Flair + T1 + T1CE modality. These plots confirm the model’s convergence and training stability. For survival classification, Figure 14 illustrates the confusion matrices for training and testing phases across three survival categories.The results show clear diagonal concentration, indicating reliable predictions. Among the modalities, F + T1 + T1CE and F + T1 + T2 + T1CE exhibit improved generalization in the test set, especially in distinguishing medium and long survival classes, which are typically more challenging. These results highlight the advantage of incorporating multimodal information for both grading and survival analysis.

Table 9 provides a comprehensive comparison of various models developed for overall survival (OS) prediction in glioma patients. Traditional machine learning models such as Gradient Boosted Trees, Random Forests, and XGBoost have reported modest performance, with accuracies ranging from 52.3% to 67.9%. More recent deep learning and ensemble-based approaches, including artificial neural networks and support vector regression, show improved results, achieving up to 86% accuracy.

A critical comparison with existing approaches is presented in Table 9, and it is important to contextualize their respective strengths and limitations. Classical machine learning–based survival models, such as Gradient Boosted Decision Trees [32] and Naive Bayes [33], are computationally efficient and interpretable, but their predictive performance is generally modest, often ranging between 52–80%. Deep learning methods, including CANet [34], leverage spatial and multimodal context to capture richer tumor characteristics; however, they come with substantial computational costs, risk of overfitting in high-dimensional settings, and reduced interpretability. Radiomics-based pipelines, such as those by [35,36,37], have shown notable improvements by exploiting handcrafted and location-related features, but their reliance on complex feature engineering and sensitivity to cohort variations can limit generalizability. Hybrid approaches that integrate segmentation with survival prediction, such as those in [38,39], improve predictive robustness but also increase system complexity and resource requirements. It is worth noting that all the compared methods, including our proposed model, were evaluated on the BraTS dataset, ensuring a fair and consistent benchmarking of performance.

**Table 9:** Comparison of the DuelContextAttn DQN model with state-of-the-art methods for overall survival prediction.

Literature Methods	OS Prediction Models	Accuracy (%)
Guo et al. [32]	Gradient Boosted Decision Tree	52.30
Pei et al. [34]	Context-Aware deep neural network (CANet)	58.60
Osman [35]	Ensemble ML models	57.80
Sun et al. [38]	Random Forest	61.00
Shboul et al. [39]	XGBoost	67.90
Soltani et al. [36]	Artificial Neural Network	78.00
Cepeda et al. [33]	Naive Bayes	80.00
Sanghani et al. [37]	Support Vector Regression	86.00
**Proposed**	**DuelContextAttn DQN**	**93.82**

The proposed DuelContextAttn DQN model significantly outperforms existing methods with an accuracy of 93.82%, demonstrating its superior ability to capture complex patterns in multimodal data. By integrating deep reinforcement learning with context-aware attention mechanisms and optimized radiomics feature selection, the model effectively enhances OS prediction performance.

### 3.3. Abalation Study

The ablation studies were conducted to evaluate the effectiveness of different feature selection strategies and reinforcement learning architectures. The analysis focuses on identifying the most efficient metaheuristic algorithm for feature selection and the most robust DQN variant for classification.

**Figure 15:**
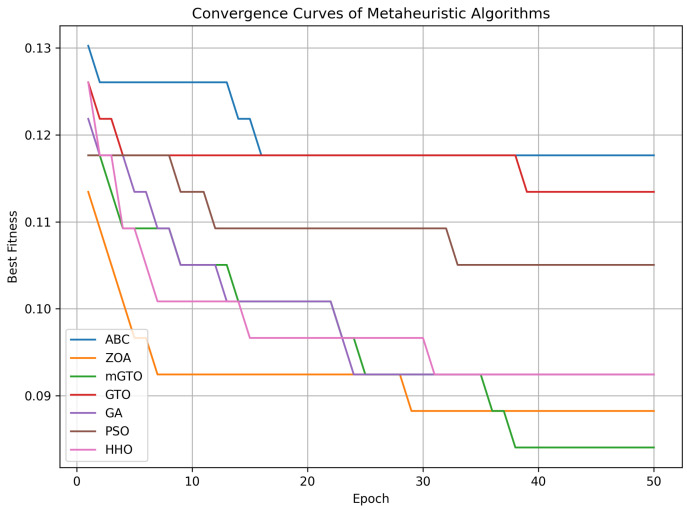
Convergence curves of HHO, ZOA, and mGTO compared with GA, PSO, and ABC, showing faster convergence and better fitness.

HHO, ZOA, and mGTO were chosen because they are recent, high-performance metaheuristics shown to offer superior exploration–exploitation balance in complex, high-dimensional feature selection tasks. In contrast, classical methods such as GA, PSO, and ABC, while well established, often suffer from premature convergence or require extensive parameter tuning. Employing these newer algorithms enabled benchmarking against traditional approaches and demonstrated clear improvements in convergence speed and final fitness, as illustrated in Figure 15.

An ablation study was performed to systematically evaluate the contribution of different architectural components to the final model’s performance. This analysis involved a comparison of six DQN variants, such as the standard DQN baseline, Double DQN, Dueling DQN, Dueling Double DQN, Dueling Double DQN with attention, and the final proposed model, DuelContextAttn DQN. All models were trained with the fused F + T1CE modality using identical splits, optimizer, schedule, replay buffer, target updates, and exploration policy. As summarized in Table 10, performance shows a monotonic improvement from DQN to the proposed DuelContextAttn DQN. Notably, while all models achieved good accuracy, the DuelContextAttn DQN obtained the highest accuracy, demonstrating the complementary benefit of incorporating context-weighted attention. These results highlight the incremental advantage of each architectural refinement, with the context-aware attention block contributing the most significant gain beyond the dueling and double Q-learning extensions.

## 4. Conclusions

GlioSurvQNet is a reinforcement learning-based framework that combines ensemble metaheuristic optimization with SHAP feature selection, modality fusion, and the DuelContextAttn DQN model for brain tumor classification and survival prediction. In the classification task, dual-modality combinations showed strong performance, with FLAIR + T1CE achieving the highest accuracy of 99.27%, demonstrating excellent discriminative power between LGG and HGG. In the survival classification task, the triple-modality fusion of FLAIR + T2 + T1CE yielded the best accuracy of 93.82%, underscoring the effectiveness of multimodal integration in enhancing predictive performance. This comprehensive strategy significantly enhances both performance and reliability, with potential benefits for clinical decision-making and patient outcomes. By leveraging reinforcement learning, the DuelContextAttn DQN continuously refines its decision-making through interaction with the environment, improving generalization and classification accuracy. Its adaptability makes it a scalable and robust solution for neuro-oncology. While GlioSurvQNet enables continuous learning, it lacks dynamic adaptation to evolving medical knowledge and treatment shifts. Moreover, its real-world applicability is constrained by reliance on a single dataset, absence of prospective clinical testing, and the need for enhanced robustness and explainability before clinical integration. It currently performs discrete survival classification; extending it to predict continuous survival times could improve clinical relevance but adds complexity. Future work includes enabling end-to-end learning from raw images and validating the model on large, multi-institutional datasets to assess real-world generalizability.

## Figures and Tables

**Table 1:** Number of radiomics features extracted from each category.

Feature Category	Count
Shape-Based Features	14
First-Order Statistics	18
GLCM (Gray Level Co-occurrence Matrix)	24
GLDM (Gray Level Dependence Matrix)	13
GLRLM (Gray Level Run Length Matrix)	16
GLSZM (Gray Level Size Zone Matrix)	15
NGTDM (Neighboring Gray Tone Difference Matrix)	5
Total	105

**Table 2:** Hyperparameters and Objective Function Settings for Metaheuristic Feature Selection.

Parameter	Value
Population Size	20
Number of Iterations	50
Optimization Type	Minimization
Search Space	Binary {0,1} over 105 features
Fitness Function	1 − accuracy_score
Classifier	SVM
Cross-validation	Stratified *k*-fold
Runs per Algorithm	10 (average accuracy reported)
Random Seeds	Different seeds for each run

**Table 3:** Training parameters of the DuelContextAttn DQN model.

Parameter	Value
State size	Number of patients cases (one state per patient sample)
Action size	2 (Classification), 3 (Survival Prediction)
Gamma (Discount Factor)	0.99
Epsilon (Initial Exploration Rate)	0.5
Epsilon_min	0.01
Epsilon_decay	0.995
Episodes	50
Batch size	16
Optimizer	Adam
Learning rate	0.001
Loss function	Mean Square Error

**Table 4:** Performance comparison with different hyperparameter settings.

Gamma	Epsilon	Epsilon_min_	Epsilon_decay_	Avg. Time (s)	Accuracy
0.99	0.5	0.0001	0.0001	1392.05	0.58
0.95	1.0	0.01	0.995	1260.33	0.92
0.90	0.7	0.05	0.01	684.37	0.94
0.85	0.3	0.1	0.003	732.56	0.94
0.99	1.0	0.01	0.995	654.74	0.98
0.99	0.5	0.01	0.995	617.26	0.99

**Table 5:** Classification Performance of DualContextAttn DQN with 5-Fold Cross-Validation.

Modality	Precision	Recall	F1-Score	Accuracy (%)
**Single-Modality **				
Flair (F)	0.99 ± 0.00 (0.99–0.99)	0.95 ± 0.01 (0.94–0.96)	0.97 ± 0.00 (0.97–0.97)	98.76 ± 0.10 (98.68–98.84)
T1	0.97 ± 0.00 (0.97–0.97)	0.96 ± 0.01 (0.95–0.97)	0.94 ± 0.01 (0.93–0.95)	**99.02 ± 0.08 (98.95–99.09) **
T2	0.97 ± 0.00 (0.97–0.97)	0.95 ± 0.01 (0.94–0.96)	0.94 ± 0.01 (0.93–0.95)	98.94 ± 0.10 (98.86–99.02)
T1CE	0.96 ± 0.01 (0.95–0.97)	0.94 ± 0.01 (0.93–0.95)	0.95 ± 0.00 (0.95–0.95)	98.50 ± 0.08 (98.42–98.58)
**Dual–Modality**				
F + T1	0.99 ± 0.00 (0.99–0.99)	0.94 ± 0.01 (0.93–0.95)	0.96 ± 0.01 (0.95–0.97)	98.53 ± 0.04 (98.49–98.57)
F + T2	0.98 ± 0.00 (0.98–0.98)	0.92 ± 0.01 (0.91–0.93)	0.95 ± 0.01 (0.94–0.96)	99.12 ± 0.08 (99.04–99.20)
F + T1CE	0.99 ± 0.00 (0.99–0.99)	0.95 ± 0.01 (0.94–0.96)	0.96 ± 0.01 (0.95–0.97)	**99.27 ± 0.05 (99.22–99.32)**
T1 + T2	0.98 ± 0.00 (0.98–0.98)	0.97 ± 0.01 (0.96–0.98)	0.97 ± 0.00 (0.97–0.97)	94.33 ± 0.08 (94.26–94.40)
T1 + T1CE	0.96 ± 0.01 (0.95–0.97)	0.96 ± 0.01 (0.96–0.97)	0.96 ± 0.00 (0.96–0.96)	97.69 ± 0.05 (97.64–97.74)
T2 + T1CE	0.99 ± 0.00 (0.99–0.99)	0.96 ± 0.01 (0.95–0.97)	0.97 ± 0.00 (0.97–0.97)	98.95 ± 0.05 (98.90–99.00)
**Triple–Modality**				
F + T1 + T2	0.97 ± 0.01 (0.97–0.99)	0.93 ± 0.01 (0.92–0.94)	0.95 ± 0.00 (0.95–0.95)	98.53 ± 0.04 (98.49–98.57)
F + T1 + T1CE	0.99 ± 0.00 (0.99–0.99)	0.96 ± 0.00 (0.96–0.96)	0.97 ± 0.00 (0.97–0.97)	**98.95 ± 0.03 (98.92–98.98)**
F + T2 + T1CE	0.98 ± 0.00 (0.98–0.98)	0.94 ± 0.01 (0.92–0.95)	0.95 ± 0.01 (0.94–0.96)	98.74 ± 0.05 (98.69–98.79)
T1 + T2 + T1CE	0.98 ± 0.00 (0.98–0.98)	0.91 ± 0.01 (0.90–0.92)	0.94 ± 0.01 (0.93–0.95)	97.69 ± 0.05 (97.64–97.74)
**All Four Modalities**				
F + T1 + T2 + T1CE	0.99 ± 0.00 (0.99–0.99)	0.91 ± 0.01 (0.90–0.93)	0.95 ± 0.00 (0.95–0.95)	98.32 ± 0.05 (98.27–98.37)

Bold highlights the highest results.

**Table 6:** Performance comparison across different models.

Model	Precision	Recall	F1-Score	Accuracy (%)
Logistic Regression	0.92	0.88	0.91	90.55
Linear SVM	0.95	0.93	0.94	94.12
XGBoost	0.96	0.95	0.95	95.31
LightGBM	0.97	0.94	0.95	96.18
Random Forest	0.95	0.92	0.93	94.88
Gradient Boosting	0.96	0.93	0.94	95.07
MLP	0.97	0.96	0.94	95.51
Proposed Method	0.99	0.94	0.96	99.27

**Table 7:** Comparative accuracy of existing models and the proposed method.

Method	Model	Accuracy (%)
Cho et al. [24]	Random Forest	88.70
Kumar et al. [25]	Random Forest	97.48
Varghese et al. [26]	SVM	97.00
Uvaneshwari et al. [27]	XGBoost	97.83
Khan et al. [28]	VGG	94.06
Rehman et al. [29]	CNN	98.32
Ferdous et al. [30]	LCDEIT	93.69
Montaha et al. [31]	TD-CNN-LSTM	98.90
Stember et al. [7]	DQL-TD	100.00 (200 episodes)
**Proposed Method**	**DuelContextAttn DQN**	**99.27 (50 episodes)**

**Table 8:** Performance of the DuelContextAttn DQN model for the Overall Survival Prediction.

Modality	Precision	Recall	F1-Score	AUC	Accuracy (%)
**Single-Modality**					
Flair	0.96	0.94	0.95	0.95	91.08
T1	0.91	0.95	0.92	0.94	93.03
T2	0.93	0.91	0.90	0.92	92.19
T1CE	0.92	0.94	0.95	0.96	**93.28**
**Dual-Modality**					
Flair + T1	0.95	0.91	0.93	0.94	91.92
Flair + T2	0.96	0.94	0.93	0.96	**93.71**
Flair + T1CE	0.93	0.91	0.90	0.94	92.76
T1 + T2	0.92	0.93	0.94	0.94	92.96
T1 + T1CE	0.90	0.91	0.91	0.91	90.76
T2 + T1CE	0.92	0.94	0.91	0.94	91.60
**Triple-Modality**					
Flair + T1 + T2	0.92	0.93	0.92	0.95	91.24
Flair + T1 + T1CE	0.96	0.91	0.94	0.95	**93.82**
Flair + T2 + T1CE	0.95	0.92	0.93	0.94	92.08
T1 + T2 + T1CE	0.94	0.92	0.93	0.94	91.71
**All Four Modalities**					
Flair + T1 + T2 + T1CE	0.93	0.94	0.95	0.95	93.39

Bold highlights the highest results.

**Table 10:** Ablation study comparing different DQN variants on the F + T1CE modality.

Model	Precision	Recall	F1-Score	Accuracy (%)
DQN	0.95	0.96	0.95	95.10
Double DQN	0.97	0.98	0.96	97.32
Dueling DQN	0.97	0.96	0.98	97.76
Dueling Double DQN	0.98	0.96	0.98	97.95
Dueling Double DQN-A	0.98	0.96	0.98	98.68
**DuelContextAttn DQN (Proposed)**	**0.99**	**0.97**	**0.98**	**99.27**

## Data Availability

The BraTS2020 dataset used for this study is publicly available at https://www.med.upenn.edu/cbica/brats2020/data.html accessed on 28 August 2025.

## Supplementary Materials

The following supporting information can be downloaded at: https://www.mdpi.com/article/10.3390/diagnostics15182304/s1, A full IBSI-compliant YAML configuration file.
