# Supplementary material for: GlioSurvQNet: A DuelContextAttn DQN Framework for Brain Tumor Prognosis with Metaheuristic Optimization
Source: Diagnostics (Basel). 2025 Sep 11;15(18):2304. doi: 10.3390/diagnostics15182304 (PMC12468973; doi:10.3390/diagnostics15182304)
Supplement: Supplementary file 1 [file diagnostics-15-02304-s001.zip › diagnostics-3802630-supplementary.pdf]

```yaml
radiomics_extraction:
  software:
    name: PyRadiomics
    version: 3.1.0
  preprocessing:
    intensity_normalization:
      method: z-score
      scope: per_volume
      mask: brain_mask
    resampling:
      voxel_size: [1, 1, 1]   # in mm
      interpolator: B-spline
  discretization:
    method: fixed_bin_width
    bin_width: 25
  roi_definition:
    source: BraTS annotations
    regions:
      - WT   # Whole Tumor
      - TC   # Tumor Core
      - ET   # Enhancing Tumor
```
